# Genome sequences of distinct genotypes of bacterial pathogen Xanthomonas euvesicatoria pv. euvesicatoria from pepper (Capsicum annuum L.) in Serbia

**DOI:** 10.1099/acmi.0.001138.v4

**Published:** 2026-02-04

**Authors:** Tatjana Popović Milovanović, Shannon F. Greer, Renata Iličić, Aleksandra Jelušić, Daisy Bown, Murray Grant, Joana G. Vicente, David J. Studholme

**Affiliations:** 1 Institute for Plant Protection and Environment, Belgrade, Serbia; 2 School of Life Sciences, University of Warwick, Coventry, UK; 3 Faculty of Agriculture, University of Novi Sad, Novi Sad, Serbia; 4 Institute for Multidisciplinary Research, University of Belgrade, Belgrade, Serbia; 5 Fera Science Ltd, York, UK; 6 Biosciences, University of Exeter, Exeter, UK

**Keywords:** *Capsicum annuum* L., genome sequence, Illumina sequencing, pathogenicity, pepper, plasmid, Serbia, Unicycler assembly, *Xanthomonas campestris *pv. *vesicatoria *group A, *Xanthomonas euvesicatoria* pv. *euvesicatoria*

## Abstract

This Technical Resource presents genome sequence data for three strains of the bacterial pathogen *Xanthomonas euvesicatoria* pv. *euvesicatoria* (*Xeu*) collected in Serbia. We isolated these strains from pepper crops showing bacterial spot symptoms in 2016 at the municipality of Irig, in the Srem district. The presented data comprise raw sequencing reads and annotated, contig-level genome assemblies. We checked for the presence of sequences of known type-3 secretion system (T3SS) effector genes and plasmid-like sequences. Phylogenomic reconstruction revealed that the three strains fell in the same clade within *Xeu*. Strain X13 is most closely related to strain 66b, collected in Bulgaria in 2012. Strains X22 and X31 are most closely related to Tu-10 collected in the Southeastern Anatolia region of Türkiye in 2020. In common with other members of the clade, all three strains share a 75 kb plasmid that carries T3SS effector genes *avrBs3*, *xopBA*, *xopAQ* and *xopE*. Additionally, strain X13 shares extensive sequence similarity to the pXCV183 plasmid, including T3SS effector gene *xopAX*, and shares extensive sequence similarity with plasmid pXap41, including T3SS effector gene *xopE3*. This difference in plasmid content might contribute to the observed difference in virulence among the Serbian *Xeu* strains. The three Serbian strains lack a 31 kb plasmid, pLMG730.4, that is seen in several Vietnamese and Canadian strains within this clade of *Xeu*. The data presented will be a useful resource for future molecular epidemiology and genomic surveillance of this pathogen in the Balkan region, augmenting the previously available draft genome sequences of *Xeu* strains 66b (Bulgaria) and 83M (North Macedonia).

## Data Summary

All genome sequence data generated in this study are publicly available via BioProject: PRJNA985260 (https://www.ncbi.nlm.nih.gov/bioproject/?term=PRJNA985260).

The raw sequence reads are deposited in the Sequence Read Archive under accession SRP444629 (https://trace.ncbi.nlm.nih.gov/Traces/?view=study&acc=SRP444629):

- SRR24958752 (strain X13)
- SRR24958751 (strain X22)
- SRR24958750 (strain X31)

All command line code is deposited in GitHub at https://github.com/davidjstudholme/xanthomonas-euvesicatoria-serbia.

The GitHub repository is also archived on Zenodo at https://doi.org/10.5281/zenodo.17194555.

## Introduction

Among European countries, Serbia is among the leaders in the production of vegetable peppers (*Capsicum annuum* L.). This encompasses diverse produce, including bell peppers and traditional varieties like Leskovac red pepper, Somborska Babura, Šorokšari, Palanačko čudo, Strižanka, Romana, Kurtovska kapija, Šilja and Slonovo uvo. Peppers are a staple in Serbian cuisine and used in a wide range of dishes, including the popular ajvar (a pepper-based relish), stuffed peppers and various salads and stews. Serbia also exports peppers and pepper products, contributing to the country’s export revenues [1]. Several diseases impact pepper production, including bacterial spot, caused by four taxa: *Xanthomonas euvesicatoria* pv. *euvesicatoria* (*Xeu*), *Xanthomonas euvesicatoria* pv. *perforans*, *Xanthomonas hortorum* pv. *gardneri* and *Xanthomonas vesicatoria* [2,6]. *Xeu*, previously known as *Xanthomonas campestris* pv. *vesicatoria* group A [7,8], is a seedborne pathogen that is listed as an A2 (high-risk) quarantine organism by the European and Mediterranean Plant Protection Organization. Besides Serbia, the *Xeu* pathogen has also been reported in Bulgaria, Czechia, Greece, Montenegro, North Macedonia and Türkiye [6,9,11]. Recently, *Xeu* was detected on pepper leaves of the cultivar (cv.) Somborka, grown in commercial fields in Kosovo [12].

Infected seeds and planting material are the primary sources of inoculum, but the pathogens can also survive in plant debris, soil and the rhizosphere of non-hosts [6]. A severe outbreak of bacterial spot disease on pepper (cv. Salcalik) in the eastern Mediterranean region of Türkiye during 1999–2002 was due to the use of infected seeds for sowing, with the disease incidence ranging from 50 to 95% [9]. In the agroecological conditions of Serbia, *Xeu* is regularly present, with the severity of disease and the extent of economic damage strongly influenced by prevailing weather conditions [13,14]. Since the mid-1980s, the disease has been steadily spreading throughout the country and is considered one of the most economically important diseases of pepper. Particularly severe outbreaks were reported in 1987, causing substantial yield losses in the Srem and Bačka regions (Vojvodina) [13,14].

Members of the genus *Xanthomonas* grow optimally between 25 and 30 °C, such that conditions in southern Europe are highly conducive to bacterial spot disease [6]. Bacteria infect host plants mainly through stomata and hydathodes but can also enter via wounds caused by mechanical damage, insect activity or adverse weather conditions [6]. Bacterial spot symptoms on pepper include angular lesions that later become brown and necrotic on the leaves, fruits, petioles and stems [6]. On leaves, small water-soaked lesions initially develop, with a greasy appearance and irregular shape. Over time, the tissue in the centre of these lesions turns brown. The spots enlarge and coalesce, and necrosis spreads over larger areas. Infected fruits develop small, greenish-brown spots that later necrotize and crack, resulting in a blistered appearance. According to the European Food Safety Authority, although recent data on economic losses caused by bacterial spot of tomato and pepper in the EU are lacking, these pathogens are considered significant for both these crops, with infections reported to cause yield losses of up to 30% [15].

Genomics has informed numerous research studies on *Xanthomonas* pathogens, including *X. euvesicatoria* [16,20]. For example, genome sequences have facilitated the cataloguing of virulence effectors [21,24], which the pathogen secretes and translocates into the host via the type-3 secretion system (T3SS). The specific repertoire of T3SS effectors influences the interactions between host and pathogen, for example, determining host range [25,26]. Genome sequences have also been crucial in developing tools for pathogen detection and for molecular epidemiology [27,30]. Although two strains of *Xeu* from neighbouring countries, North Macedonia and Bulgaria, have been sequenced [31], no genome sequence data were available for *Xeu* isolated in Serbia. Therefore, we performed genome sequencing on three strains from our collection, originating from pepper grown at the Irig locality in Serbia. The objectives were to ascertain the degree of genetic variation among the isolates and to identify which lineages of *Xeu* they belong to. Furthermore, we previously observed that isolate X13 was more virulent than strain X22 [32]; therefore, in the present study, we generated genome sequences as a resource to identify genes underlying differences in virulence among strains.

## Methods

### Isolation of bacterial strains

We isolated three strains of *Xeu* from diseased pepper leaves (cv. Crveno Zlato) showing bacterial spot symptoms in 2016 at Irig, in the Srem region of Vojvodina, Serbia. Before isolation, the leaves were first rinsed under tap water and air-dried on filter paper. Small sections were then taken from the margins of necrotic and healthy tissue and macerated in sterile distilled water. Yeast extract-dextrose-calcium carbonate (YDC) agar was used for isolation and maintenance of pure cultures. Bacterial strains develop characteristic pale yellowish, convex and mucoid bacterial colonies at 26 °C after 72 h. The strains were designated as X13 (WHRI 10185), X22 (WHRI 10186) and X31 (WHRI 10187) and confirmed to belong to *Xeu* using two conventional duplex PCRs, each including two primer combinations: Bs-XeF/Bs-XeR and Bs-XvF/Bs-XvR; Bs-XgF/Bs-XgR and Bs-XpF/Bs-XpR, as described by Koenraadt and colleagues [33].

### Pathogenicity assays

Pepper plants were grown in the pots filled with a substrate composed of white and black peat mixed in a 1:1 ratio (by volume), with a particle size of <50 mm (Domoflor, Vilnius, Lithuania). The substrate’s nutrient composition was NPK=14:16:18, with a water-holding capacity of 300–700% and a pH range of 6±1. Each pot was filled to a final weight of 5±0.5 kg and weighed daily. Pathogenicity tests were performed on young pepper plants in phase BBCH 29 (the first secondary apical lateral shoot was visible) grown under normal watering (every second day) and fertilization (1% solution of Solugal Universal fertilizer NPK 20:20:20+MNE - Anorel NV, Belgium). Bacterial suspensions were prepared by suspending pure culture of strains grown on yeast-dextrose-CaCO_3_ (YDC) agar for 48 h in sterile distilled water to an ~107 c.f.u. ml^−1^. Plants were spray-inoculated using a handheld sprayer and then covered with plastic bags to maintain high humidity for 48 h after inoculation and maintained at temperature 25±2 °C for the subsequent 10 days, after which the plants were transplanted into soil and maintained under field conditions (locality Belgrade). Pepper plants inoculated with sterile distilled water served as untreated controls. Pathogenicity testing was performed with 4 independent replicates, with each replicate containing 3 plants (i.e. a total of 12 plants per strain).

We evaluated the appearance of symptoms in the form of spots/lesions with haloes above the leaf surface during 21 days of post-inoculation period. Disease severity was rated 21 days after inoculation, using a modified 0–4 rating scale (0: symptomless leaves; 1: limited number of lesions affecting up to 10% of the leaf area; 2: leaves with 11–49% of their surface affected; 3: 50–80% of the surface affected; 4: leaves with more than 80% of the surface covered by lesions or leaves completely dead) [32]. Scores were used to calculate the disease severity index (DSI) [32], which was subjected to ANOVA and compared by Fisher’s least significant difference (LSD) test, with *P*<0.05 considered statistically significant. A boxplot analysis was generated to illustrate the variation in DSI among the strains.

Statistical analysis and visualization were conducted using R (version 4.4.3) via RStudio (version 2026.01.0+392) with packages tidyr, ggplot2 and agricolae [34,37].

To verify that the symptoms were caused by the inoculated bacteria, we re-isolated bacteria, checked colony morphology on yeast-dextrose-CaCO_3_ (YDC) medium and verified using the Bs-XeF/Bs-XeR-specific PCR primers using the methodology previously described [38].

### Extraction of genomic DNA from bacteria

Bacterial strains were grown in King’s B liquid medium at 28 °C, and DNA was extracted as described previously at protocols.io [39]. Genomic DNA was extracted from frozen bacterial pellets using the protocol described at protocols.io [40] with the Qiagen 67563 MagAttract HMW DNA Kit.

### Genomic DNA sequencing

Genomic DNA was checked by agarose gel electrophoresis and quantified using a Qubit fluorometer (Life Technologies). Whole-genome shotgun sequencing was performed on the Illumina NovaSeq 6000 platform to generate pairs of 151-nucleotide sequence reads at the University of Exeter Sequencing Facility using the NEBNext Ultra FS II DNA Library Preparation Kit for Illumina from New England Biolabs.

### Command lines, software versions and configuration files

All command lines and configuration files for the computational analyses in this study are available in the GitHub repository at https://github.com/davidjstudholme/xanthomonas-euvesicatoria-serbia [41] and on Zenodo at https://doi.org/10.5281/zenodo.17194555.

### Quality control of genomic sequence data

Prior to assembly, we performed filtering and trimming of the sequence reads using Trim Galore [42] version 0.6.7, which utilizes Cutadapt [43] version 3.5. The command line is provided in the GitHub repository that accompanies this manuscript. We set the -q threshold to 30.

### Genome assembly, deposition and annotation

We assembled the data *de novo* using SPAdes 4.0.0 [44] via the Unicycler v. 0.5.1 wrapper script. The command lines are provided in the GitHub repository. Genome assemblies were submitted to GenBank [45,46] via the NCBI Submission Portal [47], after which they were annotated via the NCBI Prokaryotic Genome Annotation Pipeline version 6.5 [48].

### Quality assessment of assembled genome sequences

Assembly quality was assessed using CheckM version 1.2.4 [49] with the marker set for genus *Xanthomonas*. The command lines are provided in the GitHub repository.

### Genome-based phylogenetic reconstruction

We generated an approximate maximum likelihood phylogenomic tree from genome sequence assemblies using PhaME [50] version 1.0.3 and FastTree 2 [51] version 2.1.11 as described in the protocol at protocols.io [52] and the GitHub repository [41]. The genome sequences included in the analysis are listed in Table S1, available in the online Supplementary Material. The resulting phylogenetic tree was rendered as graphics using the web-based Interactive Tree of Life (iTOL) [53] version 7.3.

### Identification of plasmid-like DNA sequences

We used several approaches to check for the presence of reference genes and plasmids in our newly sequenced genomes.

One method was to perform blastn (version 2.16.0) alignments against assembled genome sequences from within the Proksee web application [54]. The E-value threshold was 0.0001.

As a second method to identify contigs that likely represent plasmid sequences, we additionally ran Platon 1.7 using default settings (in ‘accuracy’ mode) [55] against the genome assemblies. Command lines and raw results are in the GitHub repository.

We also ran geNomad [56] version 1.11.2, using default settings in ‘end-to-end’ mode. The command lines and raw results are in the GitHub repository.

Another complementary approach was to align raw, unassembled genome sequencing reads against an assembled genome sequence using BWA-mem [57] version 0.7.18 and default parameter values and options. Prior to BWA-mem alignment, reads were first trimmed and filtered with Trim Galore as described in Quality control of genomic sequence data. The command lines are in the GitHub repository.

### Calculating average nucleotide identity

To calculate average nucleotide identity (ANI) between pairs of assembled genome sequences, we used FastANI [58] version 1.1. The command lines and raw results are provided in the GitHub repository.

### Identifying matches to T3SS effectors in genome sequences

We downloaded amino-acid sequences for predicted T3SS effectors on 13 June 2024 from the EuroXanth DokuWiki [59]. We used TBLASTN version 2.16.0 to check for the presence of each of the known T3SS effector genes listed at the EuroXanth Dokuwiki [59]. The command lines and raw results are in the GitHub repository. We used an E-value threshold of 1e^−10^.

### Identifying SNPs

We identified SNPs that discriminate among *Xeu* strains, from alignments of genomic sequence reads versus the reference genome sequence of *Xeu* strain 85-10. Alignment was performed using BWA-MEM 0.7.18 [57] with default settings, as described in the GitHub repository [41]. This is a conservative approach that avoids false-positive SNPs arising from errors in the *de novo* assembly process. Custom scripts [41] identified SNPs across the genomes where, for all strains, there is at least 10× read coverage and at least 95% consensus among the aligned reads. The command lines and raw results are in the GitHub repository.

### Visualizing genome comparisons

For comparative visualization of genomic regions, we used clinker (version 0.0.31), a Python-based framework for automated comparison and visualization of gene clusters [60]. Input genomic sequences were annotated and supplied to clinker, which conducts pairwise sequence similarity searches between genes and identifies homologous relationships across clusters. Gene clusters were aligned based on shared homologues, and visualizations depicting gene order, orientation and sequence similarity were generated using the accompanying clustermap.js library [60].

## Results and discussion

### Pathogenicity of the bacterial strains on pepper

**Fig. 1.**
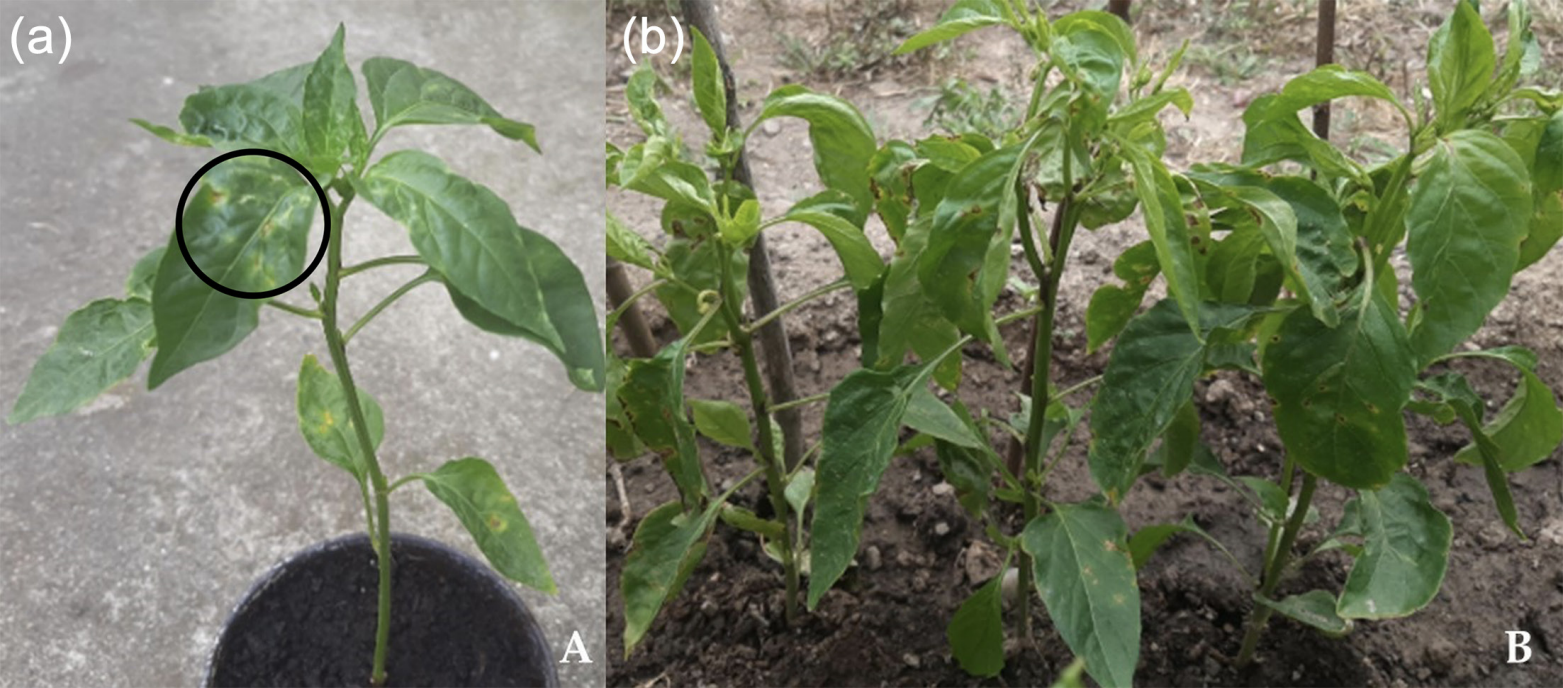
Symptoms of *Xeu* strain X13 on pepper plants. The photographs show symptoms 10 days after infection (panel a) and 21 days after infection (panel b). The black circle in panel (a) highlights an example of a necrotic lesion, surrounded by necrosis.

This study focussed on three strains from pepper crops showing bacterial spot symptoms in 2016 at the municipality of Irig, in the Srem district of Serbia. We confirmed their pathogenicity on inoculated pepper plants, forming necrotic lesions with yellow haloes 10 days post-inoculation, which progressively spread across existing and newly developed leaves over the following 3 weeks (Fig. 1).

**Fig. 2.**
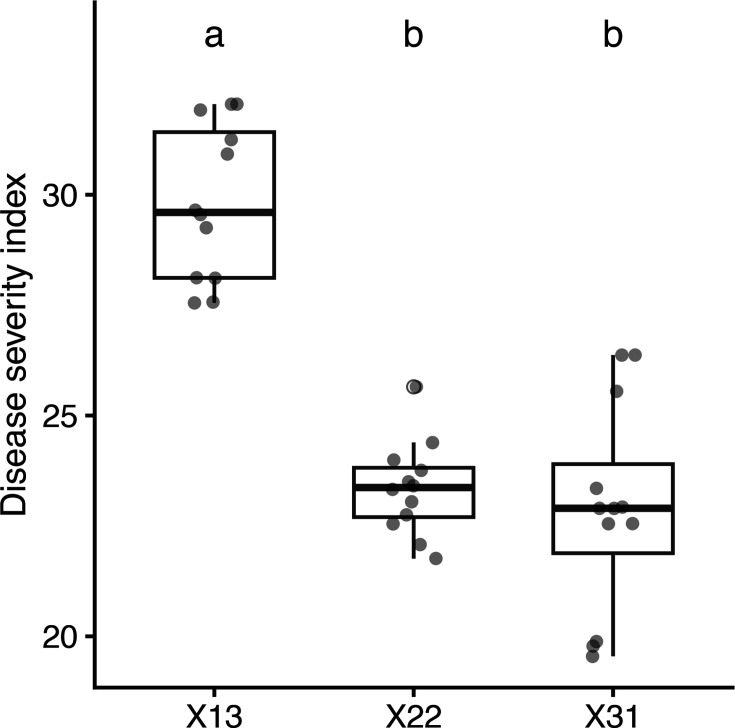
Comparison of DSI values across three groups (X13, X22 and X31). Boxplots show the median (horizontal line), interquartile range (box) and range excluding outliers (whiskers). Individual observations are overlaid as jittered points. Group differences were assessed using one-way ANOVA followed by Fisher’s LSD post-hoc test. Groups sharing the same letter are not significantly different (*P*<0.05).

Strain X13 showed higher virulence than isolates X22 and X31, as evidenced by a statistically significantly higher DSI (Fig. 2). The DSI reached 29.83% for isolate X13, while it was 23.35 and 22.89% for isolates X22 and X31, respectively. Pepper plants inoculated with sterile distilled water were symptomless.

Peppers in Serbia are predominantly cultivated from locally produced seeds, most of which belong to autochthonous varieties that have been developed and maintained over generations. Nevertheless, a smaller proportion of seeds and seedlings is imported, introducing potential variability and, possibly, new sources of *Xeu*. Tomato, as another important host for *Xanthomonas* strains causing bacterial spot, relies mainly on imported seeds, which further complicates the tracing of pathogen introduction routes. Due to these practices, combined with the wide distribution of the pathogen in neighbouring countries such as Bulgaria, Montenegro, North Macedonia and Kosovo [9,10], it remains highly challenging to pinpoint the precise time or source of its introduction into Serbian pepper and/or tomato crops. This situation highlights the need for continuous monitoring of seed health and strict phytosanitary measures to limit the spread of bacterial spot pathogens.

### Genome sequencing

Genome sequencing can discriminate close relatives and thus resolve phylogenetic relationships with high resolution [61]. We performed Illumina genome sequencing on the three *Xeu* strains X13, X22 and 31 and generated contig-level genome assemblies of between 5.1 and 5.3 Mbp (see Table 1). The numbers of contigs ranged from 76 to 98, and N_50_ was between 174.8 and 294.2 kbp. The G+C content was 64.5 mol%. We assessed the genome assemblies using CheckM, which estimates completeness by checking for the presence of single-copy marker genes that are expected to be universally present across *Xanthomonas* genomes. Our genome assemblies contained 1,159 of the 1,163 markers as single copies, and one of the markers was duplicated, with CheckM reporting a completeness score of 99.63% (see Table 1).

**Table 1.** Summary of genome sequence assembly results. Completeness and contamination were estimated using CheckM [49]. Other metrics were calculated using QUAST [70]. All genome assemblies and raw sequence data are available via BioProject PRJNA985260

Strain name	CheckM contamination score (%)*	No. of contigs	Total length of assembly (Mbp)	N_50_ length (kbp)	L_50_ count	No. of protein-coding genes	No. of tRNA	Assembly accession no.
X13 (=WHRI10185)	0.49	98	5.3	174.8	9	4619	52	GCA_030345975.2
X22 (=WHRI10186)	0.03	76	5.1	294.2	6	4150	47	GCA_030345985.2
X31 (=WHRI10187)	0.03	76	5.1	244.3	7	4153	51	GCA_030345955.2

*All three genome assemblies had a CheckM completeness score of 99.63% for the 1163 *Xanthomonas* marker set.

### Serbian *X. euvesicatoria* strains are related to strains from Bulgaria and Türkiye

**Fig. 3.**
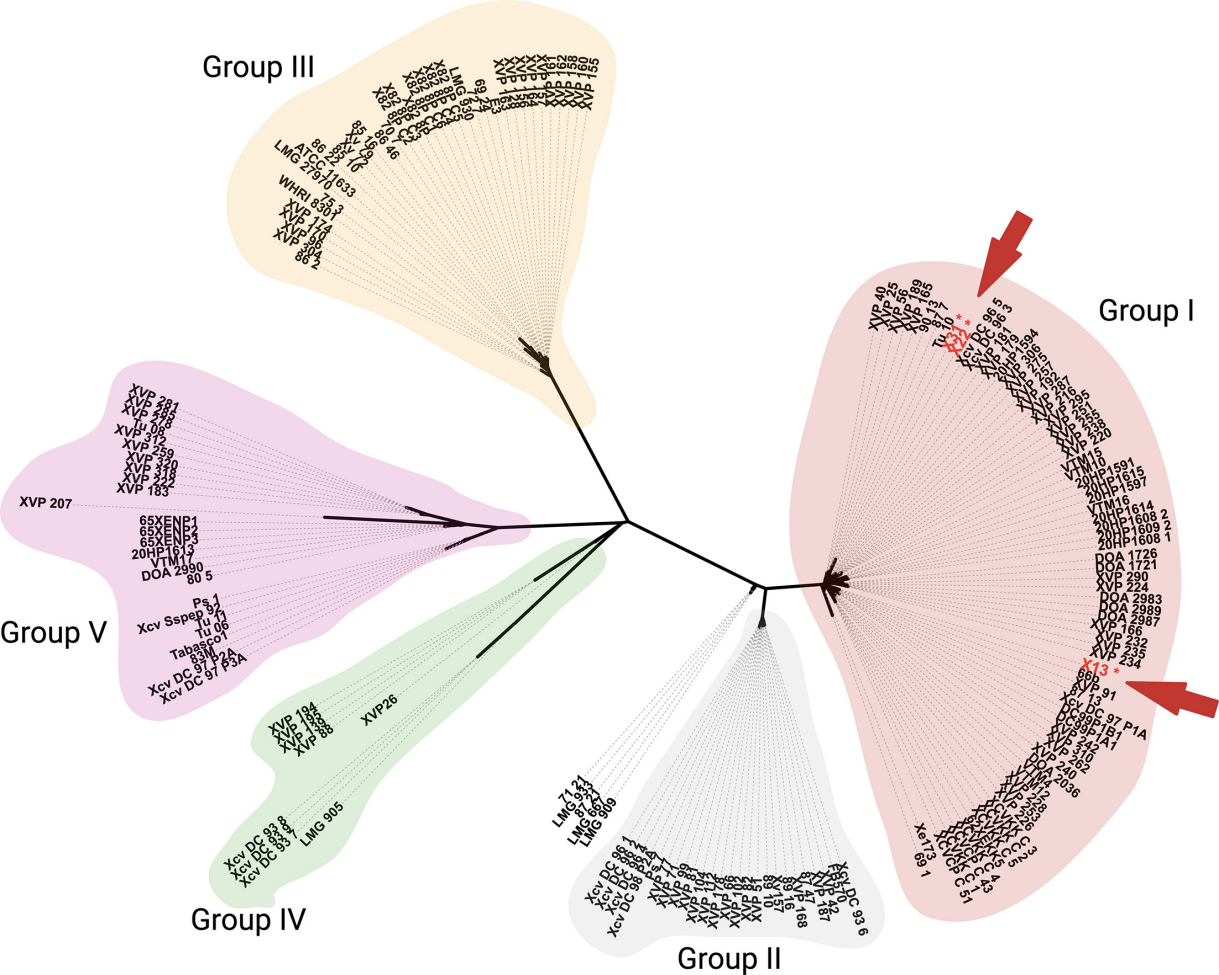
Phylogenetic placement of strains X13, X22 and X31 within *Xeu*. The tree was inferred using an approximate maximum likelihood approach implemented in FastTree 2 [51], based on the core genome alignment identified by PhaME [50]. The visualization was rendered in iTOL [53]. In addition to the three Serbian strains sequenced in this study, the analysis includes previously published genomes listed in Table S1. The major clades are shaded in different colours and named to correspond with Kaur’s previously defined groups [62]. The three Serbian strains are indicated with an asterisk (*) and red arrows. The tree is based on 13,704 SNPs distributed over a core genome length of 4,617,065 bp.

The NCBI Assembly database contains 88 genomes labelled as *Xeu* (https://www.ncbi.nlm.nih.gov/datasets/genome/?taxon=2753541 accessed on 23 December 2025). Sequenced genomes are available for strains originating in Australia, Belgium, Brazil, Canada, India, Ivory Coast, Macedonia, Taiwan, Thailand, Türkiye, Tonga, the USA and Vietnam. A recent study by Kaur and colleagues [62] found that most strains of this pathogen fall into five major clades (Groups I–V). Our phylogenomic analysis similarly revealed these five clades (Fig. 3). Our tree shares many strains in common with the phylogenetic tree previously presented by Kaur and colleagues [62], allowing cross-referencing between the clades of our tree with Kaur’s Groups I to V and hence labelling of our clades using Kaur’s nomenclature. Group III includes the type strain ATCC 11633 [12,63] and the widely used reference strain 85-10 [64].

Strains from the Balkan Peninsula fall into two clades. Specifically, strain 83M from North Macedonia [31] falls within Group V along with strains from Canada, Türkiye and Vietnam. This North Macedonian strain is particularly similar to strain DC 97 P2A [63] from Canada, possibly highlighting the capacity for this pathogen to be disseminated over long distances.

**Fig. 4.**
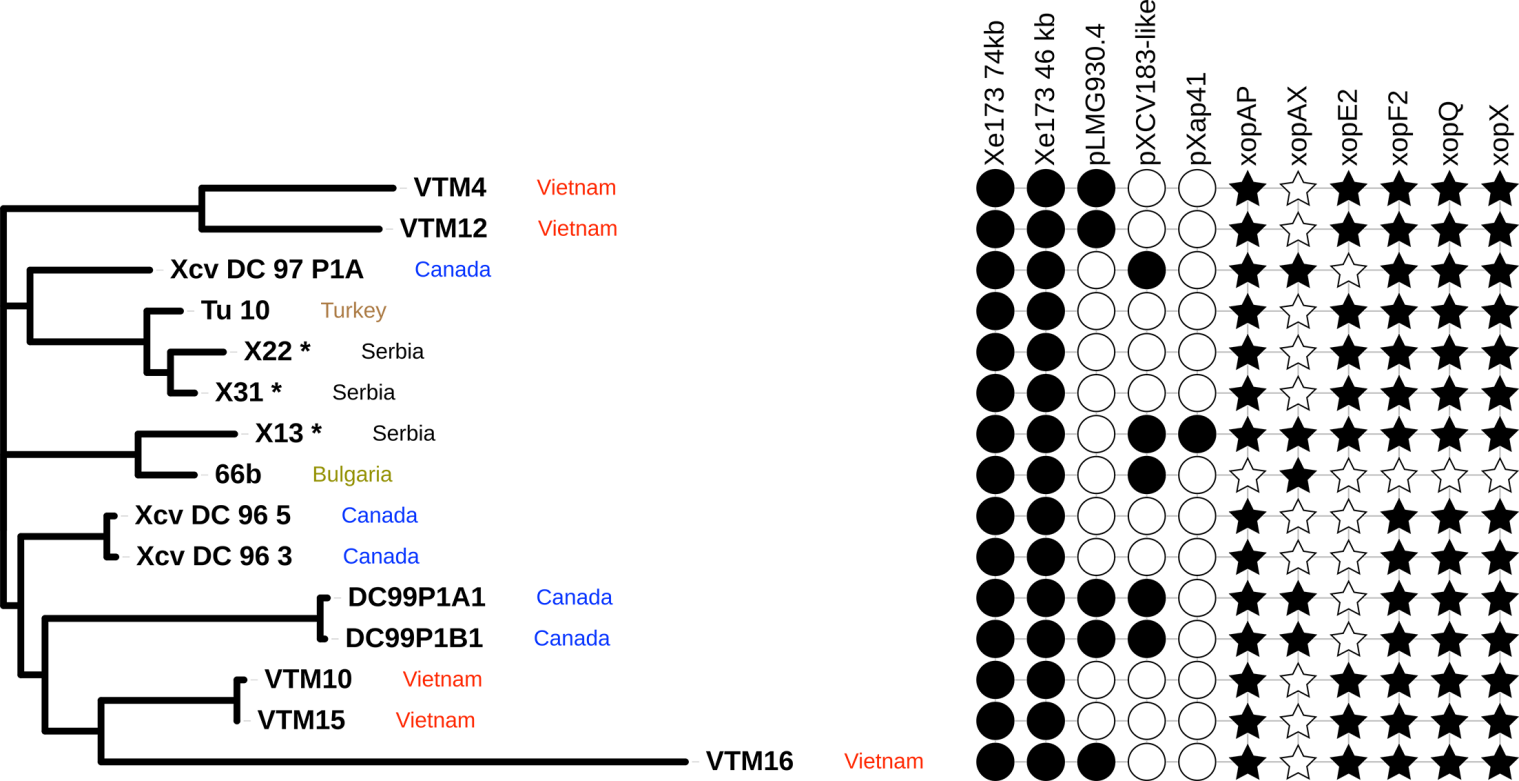
Phylogenetic position of strains X13, X22 and X31 within Group I. The approximate maximum likelihood tree is based on core genome sequences, generated using PhaME [50] and FastTree 2 [51]. In addition to the genomes of three Serbian strains, the tree also includes previously published genome sequences, which are listed in Table S1. Presence and absence of plasmid sequences are indicated by black and white circles, respectively. Presence and absence of plasmids were inferred from BWA-mem alignments of sequence reads against the relevant reference sequences, as illustrated in Figs S3–S7. Presence of effector genes in each assembled genome sequence is indicated by black stars; presence is defined as TBLASTN hit that covers at least 95% of the query effector sequences with at least 95% amino-acid sequence identity. The tree was graphically rendered using iTOL [53]. Each of the three Serbian strains sequenced in this study is marked with an asterisk (*). The tree is based on 897 SNPs distributed over a core genome length of 5,029,310 bp.

Besides strain 83M in Group V, the other Balkan strains fall into Group I. Fig. S1 shows a phylogenomic tree for members of Group I only, with other clades excluded to improve clarity. Fig. 4 shows just those strains in Group I for which genome sequence reads are publicly available. Group I includes strain 66b isolated in Bulgaria [31] in 2012, as well as the three Serbian strains, plus strains from Canada [63], Taiwan [62,63], Türkiye [65], the USA [62] and Vietnam [66]. Within this clade, the Serbian strain X13 is nearly identical (99.8696% ANI) to the Bulgarian strain 66b (Fig. S1). Serbian strains X22 and X31 are nearly identical to each other (99.9983% ANI) and are located on a different sub-branch in Group I, nearly identical to strain Tu-10 isolated from the Southeastern Anatolia region of Türkiye in 2020. This pattern suggests that there may have been at least two introductions of the pathogen into the Srem region of Vojvodina (Irig), Serbia: one introduction is represented by X13 and may be epidemiologically linked to neighbouring Bulgaria, while X22 and X31 represent at least one separate introduction that may be linked to Türkiye. Based on the currently available strains and sequence data, it is not possible to determine the direction or the date of the pathogen’s incursions.

We identified SNPs in the core genome, using alignments of genomic sequence reads versus the 85-10 reference genome sequence; this approach avoids errors introduced during the *de novo* assembly process. The numbers of SNPs separating each strain are summarized in Fig. S2. Strains X22 and X31 are separated from each other by 22 SNPs, and they are separated from strain Tu-10 by 23 and 13 SNPs, respectively. Strain X13 is separated from strain 66b by 40 SNPs. These SNPs are listed in Tables S2–S5.

### Comparison against *Xeu* 85-10 reference chromosome

We compared the genomes of the three Serbian strains against the chromosome of the *Xeu* reference strain 85-10 [64] (GenBank: AM039952.1). Most of the reference chromosome is conserved in X13, X22 and X31 (see Fig. S3). However, there are several regions of the 85-10 chromosome that are not conserved in the Serbian strains.

The 25.2 kb chromosomal region spanning approximately positions 244–269 kb in the 85-10 chromosome is not conserved in the Serbian strains. This region includes loci XCV0209–XCV0226. Most of the predicted CDSs in this region are of unknown function. However, it does include putative transposase (XCV0214), IS 1477 transposase (XCV0222, XCV0223) and IS xac2 transposase (XCV0228, XCV0229), suggesting that it represents a mobile element.

**Fig. 5.**
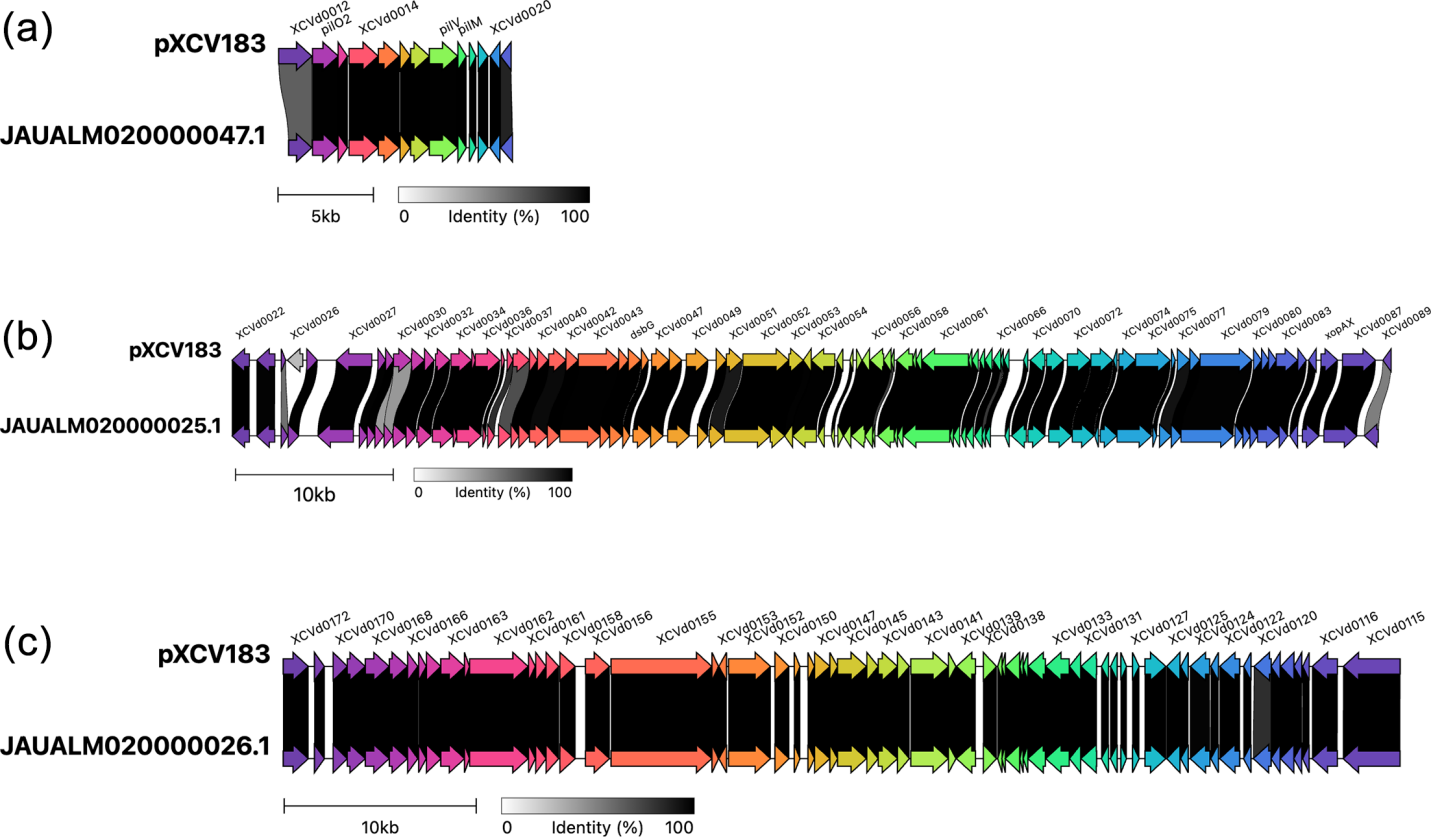
The genome of *Xeu* strain X13 contains sequences similar to plasmid pXCV183. Three contigs from the *Xeu* X13 genome assembly are each aligned against the sequence of plasmid pXCV183 [64] using clinker [60]. The GenBank accession numbers of the contigs are indicated at the left side of each contig. (a–c) Each shows sections of pXCV183 aligned to various X13 genome contigs.

The 85-10 chromosome contains a 14 kb region with homology to the filamentous phage phi-Lf at ~2,784–2,798 kb [64] and likely represents a prophage that has integrated into the bacterial genome. Part of this phage-like region of the 85-10 chromosome is missing from the Serbian strains (see Fig. S3). Also in this vicinity is a large region of the 85-10 chromosome that is not conserved in the Serbian strains, nor in other strains belonging to *Xeu* Group I. This region contains numerous genes of unknown function. It also contains at least four copies of insertion sequence IS 6100 and several genes whose functions are predicted (Fig. 5). This includes *gcvA*, *tniB*, *zitB*, *copA*, *strAB*, *qseBC* and *dsbG*.

Between the *tRNA-Thr* and *cyoA* genes in the 85-10 chromosome are two IS 1477 insertion sequences and an IS xac2 transposase gene adjacent to the *xopG* gene that encodes a T3SS system effector, XopG. This *xopG* virulence effector gene is not conserved in the three Serbian strains. This is consistent with previous studies that have shown that the chromosomal *xopG* gene is somewhat unique to strain 85-10 [67].

### Strain X13 (but not X22 and X31) contains plasmids similar to pXap41 and pXCV183

Having compared sequences against the reference strain 85-10 chromosome, we investigated whether there was any evidence of plasmids in the sequencing data for Serbian strains X13, X22 and X31. Platon identified nine contigs in X13, six contigs in X22 and five contigs in X31 that likely represent plasmid sequences; these contigs are listed in Table 2.

**Table 2.** Platon and geNomad identify contigs potentially representing plasmid sequences. Genome sequence assemblies of strains X13, X22 and X31 were searched for likely plasmid sequences using Platon [55] in the default ‘accuracy’ mode and using geNomad [56] in default end-to-end mode

**Strain X13**					
**Accession number**	**Length (bp)**	**blastn** **match name (and length)**	**blastn** **match accession number**	**Nucleotide sequence identity (%)**	**geNomad plasmid score**
JAUALM020000026.1	72,015	pXCV183	AM039951.1	99.98	0.9995
JAUALM020000047.1	11,728	pXCV183	AM039951.1	99.88	1.0000
JAUALM020000038.1	18,296	pXap41	FR875157.1	99.98	1.0000
JAUALM020000032.1	30,942	Xe173 (46 kbp)	CP170255.1	100	1.0000
JAUALM020000035.1	24,998	Xe173 (75 kbp)	CP170255.1	100	1.0000
JAUALM020000044.1	12,819	Xe173 (75 kbp)	CP170255.1	99.85	1.0000
JAUALM020000055.1	4,575	Xe173 (75 kbp)	CP170255.1	100	0.9970
JAUALM020000067.1	1,798	Xe173 (75 kbp)	CP170255.1	100	0.9999
JAUALM020000069.1	1,692	Xe173 (75 kbp)	CP170255.1	100	1.0000
**Strain X22**					
**Accession number**	**Length (bp)**	**blastn** **match name (and length)**	**blastn** **match accession number**	**Nucleotide sequence identity (%)**	
JAUALL020000023.1	30,942	Xe173 (46 kbp)	CP170255.1	100	1.0000
JAUALL020000027.1	21,713	Xe173 (75 kbp)	CP170254.1	100	1.0000
JAUALL020000035.1	10,600	Xe173 (75 kbp)	CP170254.1	100	1.0000
JAUALL020000037.1	10,214	Xe173 (75 kbp)	CP170254.1	99.81	1.0000
JAUALL020000042.1	6,087	Xe173 (75 kbp)	CP170254.1	100	1.0000
JAUALL020000048.1	2,494	Xe173 (75 kbp)	CP170254.1	100	0.8925
**Strain X31**					
**Accession number**	**Length (bp)**	**blastn** **match name (and length)**	**blastn** **match accession number**	**Nucleotide sequence identity (%)**	
JAUALK020000025.1	32,629	Xe173 (46 kbp)	CP170255.1	100	1.0000
JAUALK020000029.1	21,710	Xe173 (75 kbp)	CP170254.1	100	1.0000
JAUALK020000036.1	12,880	Xe173 (75 kbp)	CP170254.1	99.85	1.0000
JAUALK020000038.1	10,803	Xe173 (75 kbp)	CP170254.1	100	1.0000
JAUALK020000044.1	6,087	Xe173 (75 kbp)	CP170254.1	100	1.0000

blastn searches confirmed that several of the contigs identified by Platon share similarity with previously reported sequences of *Xanthomonas* plasmids; results are summarized in Fig. 4. The reference strain 85-10 contains a large plasmid pXCV183, which includes genes for the virulence effector genes *xopH*, *avrBs1* and *xopAX*. blastn alignments of the X13 genome sequence against pXCV183 revealed extensive sequence conservation of much of its length but not all (Fig. 5). There was no sequence similarity to *xopH* nor *avrBs1*, but there was conservation of *xopAX*. The X13 genome gave complete coverage and 100% sequence identity against the pXCV183-like plasmid pLMG930.2 in the genome of *Xeu* strain LMG930 [68]. This suggests that Serbian strain X13 harbours a pXCV183-like plasmid identical or nearly identical to pLMG930.2, which includes the *xopAX* gene. The other two Serbian strains (X22 and X31) showed only very limited sequence similarity to pLMG930.2, indicating that they do not share this pXCV183-like plasmid (Fig. S4).

**Fig. 6.**
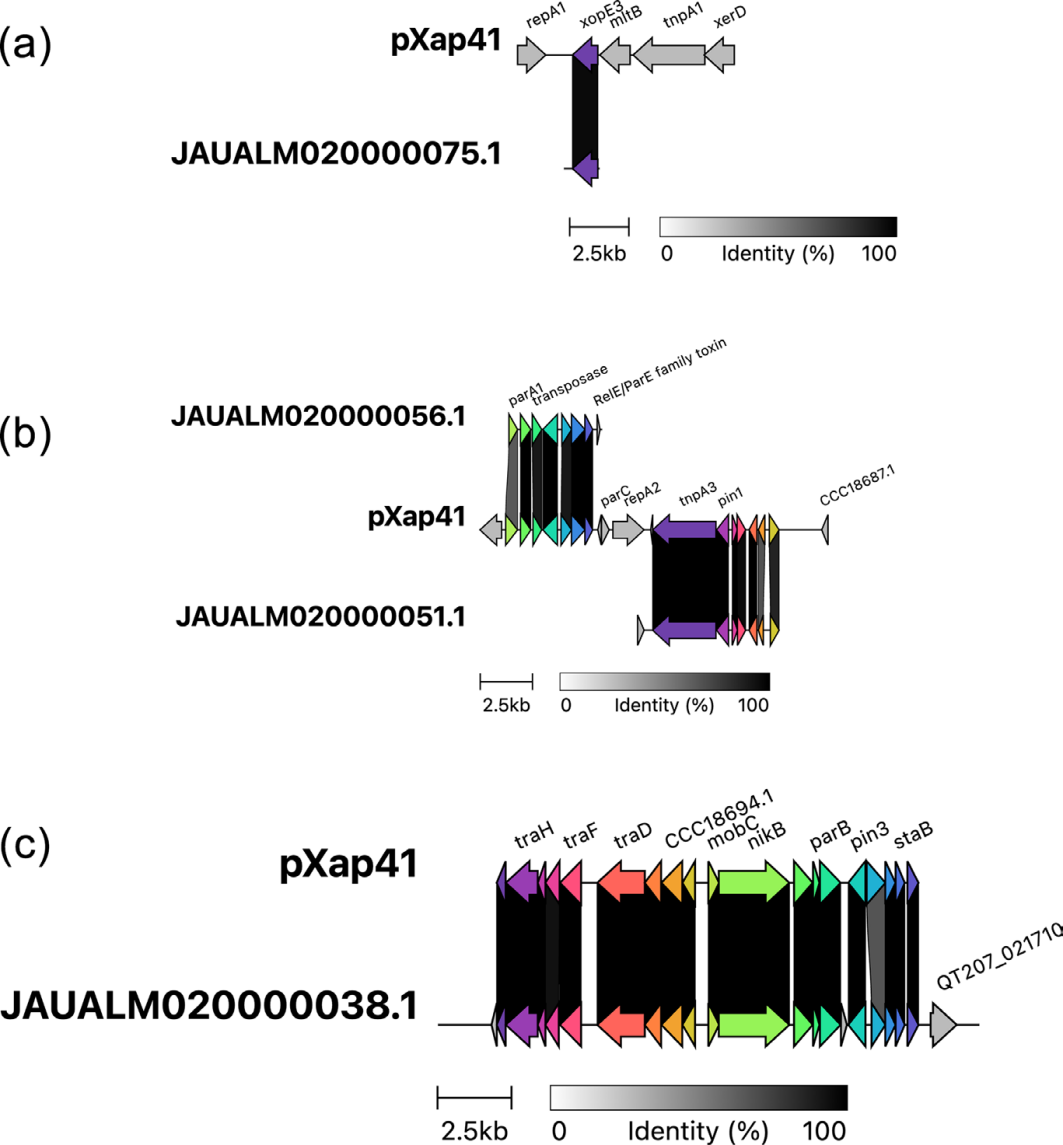
The genome of *Xeu* strain X13 contains sequences similar to plasmid pXap41. Four contigs from the *Xeu* X13 genome assembly are each aligned against the sequence of plasmid pXap41 [69] using clinker [60]. The GenBank accession numbers of the contigs are indicated at the left side of each contig. (a–c) Each shows sections of pXap41 aligned to various X13 genome contigs.

The Serbian strain X13 also shares extensive sequence conservation over most of the length of plasmid pXap41 [69] from *Xanthomonas arboricola* pv. *pruni* strain CFBP 5530 (RefSeq: NC_016053.1); see Fig. 6. This sequence conservation includes the virulence effector gene *xopE* (Fig. S5). This suggests that strain X13 contains a pXap41-like plasmid. Serbian strains X22 and X31 contain sequences that match only about half of the pXap41 plasmid, including the *xopE3* gene. This suggests that these strains might contain another distinct plasmid that shares some limited similarity with pXap41; unfortunately, the assembly of only short sequence reads precludes the complete assembly of plasmid sequences, and resolution would require future sequencing with long reads. It was previously reported that pXap41 was sufficiently unique to *X. arboricola* pv. *pruni* that it could be used for pathovar detection and identification [69]. However, its presence in at least one *Xeu* strain calls its diagnostic usefulness into question.

### Strains X13, X22 and X31 have sequences matching 75 and 46 kbp plasmids from strain Xe173

All three Serbian strains (X13, X22 and X31) showed conservation of almost the entire length of a 75 bp plasmid from Xe173, a *Xeu* strain of unknown origin, isolated from pepper and sequenced at Chonnam National University, Korea (BioSample: SAMN42326212); see Fig. S6. This plasmid harbours virulence effector genes *avrBs3*, *xopE3*, *xopAQ* and *xopBA*.

### T3SS effectors

Genomes of strains X13, X22 and X31 contained genes for all of the previously defined [18] core effectors found in *Xanthomonas* strains that infect tomato or pepper, that is, effectors AvrBs2, XopD, XopE2, XopF1, XopK, XopL, XopN, XopQ, XopR, XopX, XopZ1 and XopAD. Also present in all three genomes were genes for XopC1, XopE1, XopF2, XopI, XopJ1, XopJ3, XopO, XopP, XopAA, XopAI, XopAK and XopAP, all of which are commonly found in *X. euvesicatoria*. These genes, like the core effector genes, are located on the chromosome of reference strain 85-10 [64]. Additionally, the three Serbian strains all contain genes for AvrBs3, XopE3, XopAQ and XopBA, located on a 75 kbp plasmid. They lack the chromosomal gene for XopG.

Despite sharing most of their effector repertoires in common, there are some differences between Serbian strain X13 versus X22 and X31. Strain X13 contains a gene for XopAX, located on a pXCV183-like plasmid that is not found in strains X22 and X31.

Additionally, strain X13 shares extensive sequence similarity to the pXCV183 plasmid, including T3SS effector gene *xopAX*, and X13 shares extensive sequence similarity with plasmid pXap41, including T3SS effector gene *xopE3*. None of the three Serbian strains share the 31 kb plasmid pLMG730.4 that is seen in several Vietnamese and Canadian strains within this clade of *Xeu*.

This protein, encoded by the *xopAX* gene, is one of four novel effectors discovered in strain 85-10 by genome sequence analysis followed by a laboratory-based assay for translocation into host cells [23]. The molecular function of XopAX is unknown; it has no clear homologues or known biochemical activity. Being plasmid-encoded and patchily distributed among *Xeu* strains, XopAX is a candidate for recent horizontal transfer and could contribute to strain-specific host interactions or virulence differences [67]. Therefore, we hypothesize that the presence of the *xopAX* gene in strain X13 might contribute to its increased virulence.

## Conclusion

These data will be a useful resource for future molecular epidemiology and genomic surveillance of this pathogen in the Balkan region, augmenting the previously available draft genome sequences of *Xeu* strains 66b (Bulgaria) and 83M (North Macedonia). The genome sequences reveal that Serbian strains fall within the same clade as strain 66b and not the same clade as 83M. Although Serbian strain X13 is genetically almost identical to Bulgarian strain 66b, there are genetic differences among Serbian strains, notably in respect of plasmid-like sequences, and with strains X22 and X31 being more distantly related. These differences, e.g. in the virulence effector *xopAX*, presumably contribute to the observed difference in their virulence.

## Supplementary material

10.1099/acmi.0.001138.v4 Uncited Supplementary Material 1.

## Abbreviations

ANI: average nucleotide identity
DSI: disease severity index
iTOL: Interactive Tree of Life
LSD: least significant difference
T3SS: type-3 secretion system
*Xeu*: *Xanthomonas euvesicatoria* pv. *euvesicatoria*
YDC: yeast extract-dextrose-calcium carbonate
