# Supplementary material for: Genome sequences of distinct genotypes of bacterial pathogen Xanthomonas euvesicatoria pv. euvesicatoria from pepper (Capsicum annuum L.) in Serbia
Source: Access Microbiol. 2026 Feb 4;8(2):001138.v4. doi: 10.1099/acmi.0.001138.v4 (PMC12872329; doi:10.1099/acmi.0.001138.v4)
Supplement: Uncited Supplementary Material 1. [file acmi-8-01138-s001.pdf]

Supplementary information

Table S1. Genome sequence assemblies used in this study.

Accession number	Strain	Geographical location	Reference
GCA_020880045.1	87-47	Australia	[1]
GCA_020880235.1	Xv157	Australia	[1]
GCA_001401555.1	LMG 27970	Belgium: Merelbeke	-
GCA_001691375.1	LMG 933	Brazil	[2]
GCA_000802325.1	66b	Bulgaria: Pavlikeni, Veliko Tarnovo	[3]
GCA_020879135.1	Ps-7	Canada: Ontario	[1]
GCA_020879635.1	Xcv DC 93-9	Canada: Ontario	[1]
GCA_020879655.1	Xcv DC 97 P1A	Canada: Ontario	[1]
GCA_020879745.1	Xcv DC 96-3	Canada: Ontario	[1]
GCA_020879775.1	Xcv DC 96-1	Canada: Ontario	[1]
GCA_020879795.1	DC99P1B1	Canada: Ontario	[1]
GCA_020879815.1	Xcv DC 98 P2A	Canada: Ontario	[1]
GCA_020879825.1	Xcv DC 93-6	Canada: Ontario	[1]
GCA_020879845.1	Xcv DC 96-5	Canada: Ontario	[1]
GCA_020879875.1	Xcv DC 96-4	Canada: Ontario	[1]
GCA_020880275.1	Ps-1	Canada: Ontario	[1]
GCA_020880315.1	Xcv DC 93-8	Canada: Ontario	[1]
GCA_020880395.1	Xcv Sspep 92	Canada: Ontario	[1]
GCA_020880575.1	Xcv DC 96-2	Canada: Ontario	[1]
GCA_020880635.1	Xcv DC 97 P2A	Canada: Ontario	[1]
GCA_020880835.1	Xcv DC 93-7	Canada: Ontario	[1]
GCA_020880855.1	DC99P1A1	Canada: Ontario	[1]
GCA_020880895.1	Xcv DC 97 P3A	Canada: Ontario	[1]
GCA_001691325.1	LMG 905	India	[2]
GCA_001691345.1	LMG 909	Ivory Coast	[2]
GCA_000802345.1	83M	Macedonia: Strumitsa	[3]
GCA_030345955.2	X31	Serbia: Irig	This study
GCA_030345975.2	X13	Serbia: Irig	This study
GCA_030345985.2	X22	Serbia: Irig	This study
GCA_040055865.1	XVP-99	Taiwan	[4]
GCA_040055875.1	XVP-96	Taiwan	[4]

GCA_040055935.1	XVP-91	Taiwan	[4]
GCA_040055895.1	XVP-88	Taiwan	[4]
GCA_040055885.1	XVP-82	Taiwan	[4]
GCA_040055965.1	XVP-81	Taiwan	[4]
GCA_040056005.1	XVP-77	Taiwan	[4]
GCA_040055995.1	XVP-71	Taiwan	[4]
GCA_040056045.1	XVP-66	Taiwan	[4]
GCA_040056065.1	XVP-56	Taiwan	[4]
GCA_040056105.1	XVP-51	Taiwan	[4]
GCA_040057345.1	XVP-162	Taiwan	[4]
GCA_040057325.1	XVP-161	Taiwan	[4]
GCA_040057375.1	XVP-160	Taiwan	[4]
GCA_040057365.1	XVP-158	Taiwan	[4]
GCA_040057425.1	XVP-157	Taiwan	[4]
GCA_040057405.1	XVP-156	Taiwan	[4]
GCA_040057445.1	XVP-155	Taiwan	[4]
GCA_040057455.1	XVP-139	Taiwan	[4]
GCA_040057485.1	XVP-128	Taiwan	[4]
GCA_040055785.1	XVP-112	Taiwan	[4]
GCA_040055805.1	XVP-104	Taiwan	[4]
GCA_040055815.1	XVP-102	Taiwan	[4]
GCA_040057145.1	XVP-179	Taiwan	[4]
GCA_040057165.1	XVP-178	Taiwan	[4]
GCA_040057185.1	XVP-174	Taiwan	[4]
GCA_040057185.1	XVP-174	Taiwan	[4]
GCA_040057225.1	XVP-170	Taiwan	[4]
GCA_040057205.1	XVP-168	Taiwan	[4]
GCA_040057245.1	XVP-166	Taiwan	[4]
GCA_040057265.1	XVP-165	Taiwan	[4]
GCA_040057285.1	XVP-164	Taiwan	[4]
GCA_040057305.1	XVP-163	Taiwan	[4]
GCA_040056845.1	XVP-226	Taiwan	[4]
GCA_040056905.1	XVP-224	Taiwan	[4]
GCA_040056825.1	XVP-222	Taiwan	[4]
GCA_040056925.1	XVP-220	Taiwan	[4]
GCA_040056965.1	XVP-216	Taiwan	[4]
GCA_040056985.1	XVP-207	Taiwan	[4]
GCA_040057005.1	XVP-195	Taiwan	[4]

GCA_040057045.1	XVP-194	Taiwan	[4]
GCA_040057025.1	XVP-192	Taiwan	[4]
GCA_040057065.1	XVP-189	Taiwan	[4]
GCA_040057085.1	XVP-181	Taiwan	[4]
GCA_040057125.1	XVP-183	Taiwan	[4]
GCA_040057105.1	XVP-187	Taiwan	[4]
GCA_040056645.1	XVP-253	Taiwan	[4]
GCA_040056425.1	XVP-275	Taiwan	[4]
GCA_040056515.1	XVP-262	Taiwan	[4]
GCA_040056605.1	XVP-257	Taiwan	[4]
GCA_040056575.1	XVP-259	Taiwan	[4]
GCA_040056625.1	XVP-255	Taiwan	[4]
GCA_040056655.1	XVP-251	Taiwan	[4]
GCA_040056675.1	XVP-25	Taiwan	[4]
GCA_040056735.1	XVP-242	Taiwan	[4]
GCA_040056745.1	XVP-240	Taiwan	[4]
GCA_040056725.1	XVP-238	Taiwan	[4]
GCA_040056785.1	XVP-235	Taiwan	[4]
GCA_040056755.1	XVP-234	Taiwan	[4]
GCA_040056865.1	XVP-232	Taiwan	[4]
GCA_040056885.1	XVP-228	Taiwan	[4]
GCA_040056075.1	XVP-42	Taiwan	[4]
GCA_040056135.1	XVP-40	Taiwan	[4]
GCA_040056125.1	XVP-320	Taiwan	[4]
GCA_040056185.1	XVP-318	Taiwan	[4]
GCA_040056245.1	XVP-312	Taiwan	[4]
GCA_040056215.1	XVP-310	Taiwan	[4]
GCA_040056265.1	XVP-306	Taiwan	[4]
GCA_040056285.1	XVP-304	Taiwan	[4]
GCA_040056335.1	XVP-295	Taiwan	[4]
GCA_040056305.1	XVP-290	Taiwan	[4]
GCA_040056325.1	XVP-287	Taiwan	[4]
GCA_040056345.1	XVP-285	Taiwan	[4]
GCA_040056385.1	XVP-281	Taiwan	[4]
GCA_040056405.1	XVP-278	Taiwan	[4]
GCA_020880135.1	87-21	Taiwan	[1]
GCA_040091395.1	XVP26	Taiwan	[5]
GCA_020880015.1	89-10	Thailand	[1]

GCA_048855765.1	65XENP3	Thailand	[6]
GCA_048855785.1	65XENP1	Thailand	[6]
GCA_048855825.1	65XENP2	Thailand	[6]
GCA_048855905.1	20HP1615	Thailand	[6]
GCA_048855925.1	20HP1614	Thailand	[6]
GCA_048855965.1	20HP1613	Thailand	[6]
GCA_048855985.1	20HP1608-2	Thailand	[6]
GCA_048856005.1	20HP1609-2	Thailand	[6]
GCA_048856105.1	20HP1608-1	Thailand	[6]
GCA_048856135.1	20HP1597	Thailand	[6]
GCA_048856265.1	20HP1594	Thailand	[6]
GCA_048856285.1	62XEKK5	Thailand	[6]
GCA_048856305.1	20HP1591	Thailand	[6]
GCA_048856325.1	XCVCP-C-51	Thailand	[6]
GCA_048856365.1	XCVCP-C-43	Thailand	[6]
GCA_048856465.1	XCVKK-C-4	Thailand	[6]
GCA_048856485.1	XCVKK-C-5	Thailand	[6]
GCA_048856505.1	XCVKK-C-3	Thailand	[6]
GCA_048856545.1	XCVKK-C-2	Thailand	[6]
GCA_048856565.1	XCVKK-C-1	Thailand	[6]
GCA_048856605.1	DOA-2987	Thailand	[6]
GCA_048856625.1	DOA-2990	Thailand	[6]
GCA_048856645.1	DOA-2989	Thailand	[6]
GCA_048856725.1	DOA-2983	Thailand	[6]
GCA_048856805.1	DOA-1721	Thailand	[6]
GCA_048856825.1	DOA-2036	Thailand	[6]
GCA_048856865.1	DOA-1726	Thailand	[6]
GCA_028598835.1	Tu-08	Turkey	[7]
GCA_028598895.1	Tu-06	Turkey	[7]
GCA_028599415.1	Tu-11	Turkey	[7]
GCA_028599425.1	Tu-10	Turkey	[7]
GCA_000009165.1	85-10	Unknown	[8]
GCA_001691315.1	LMG 667 (= ICMP 4799)	Tonga	[2]
GCA_020879415.1	FB570	Unknown	[1]
GCA_042448555.1	Xe173	Unknown	[5]
GCA_001908795.1	LMG 930	USA	[9]
GCA_040091415.1	X82-8P_C6	USA	[5]

GCA_040091435.1	X82-8P_C5	USA	[5]
GCA_040091455.1	X82-8P_C4	USA	[5]
GCA_040091475.1	X82-8P_C3	USA	[5]
GCA_040091495.1	X82-8P_C2	USA	[5]
GCA_040091515.1	X82-8P_C1	USA	[5]
GCA_040091535.1	X82-8P	USA	[5]
GCA_040202735.2	WHRI 8301 (= 75-3)	USA	[1]
GCA_045763815.1	Tabasco1	USA	[5]
GCA_045763835.1	90-13	USA	[5]
GCA_045763855.1	87-7	USA	[5]
GCA_045763865.1	89-16	USA	[5]
GCA_045763895.1	87-13	USA	[5]
GCA_045763915.1	80-5	USA	[5]
GCA_045763955.1	72-7	USA	[5]
GCA_045763975.1	71-21	USA	[5]
GCA_045764005.1	70-7	USA	[5]
GCA_045764015.1	69-1	USA	[5]
GCA_045764025.1	69-24	USA	[5]
GCA_020879155.1	Xv 79	USA: Florida	[1]
GCA_020879265.1	Xv 72	USA: Florida	[1]
GCA_020879485.1	E3	USA: Florida	[1]
GCA_020880035.1	86-46	USA: Florida	[1]
GCA_020880115.1	86-22	USA: Florida	[1]
GCA_020880165.1	86-2	USA: Florida	[1]
GCA_020880345.1	71-21	USA: Florida	[1]
GCA_020880375.1	85-16	USA: Florida	[1]
GCA_020880435.1	75-3	USA: Florida	[1]
GCA_020880415.1	ATCC 11633	USA: New Jersey	[1]
GCA_033548465.1	VTM16	Vietnam	[10]
GCA_033548475.1	VTM15	Vietnam	[10]
GCA_033548515.1	VTM17	Vietnam	[10]
GCA_033548535.1	VTM10	Vietnam	[10]
GCA_033548565.1	VTM4	Vietnam	[10]
GCA_033548575.1	VTM12	Vietnam	[10]

Table S2. Single-nucleotide polymorphisms that distinguish strain X31 from strain Tu-10.

Position on GenBank: AM039952.1	Reference allele	Alternative allele	66b	**Tu-10**	X13	X22	**X31**
109416	a	g		**x**			
690978	a	g		**x**			
816441	g	t		**x**			
861129	a	c					**x**
991500	t	c		**x**			
1788346	t	g					**x**
1805242	t	c					**x**
2169416	a	g					**x**
2537946	g	a		**x**			
2934251	c	g					**x**
3205571	g	a					**x**
3839830	t	c		**x**			
4054862	t	c		**x**			

Table S3. Single-nucleotide polymorphisms that distinguish strain X22 from strain Tu-10.

Position on GenBank: AM039952.1	Reference allele	Alternative allele	66b	**Tu-10**	X13	**X22**	X31
74213	t	c				x	
109416	a	g		x			
512211	a	g				x	
690978	a	g		x			
809587	a	g				x	
816441	g	t		x			
991500	t	c		x			
995341	t	c				x	
1018308	g	c				x	
1961368	g	a				x	
2172890	a	t				x	
2387221	a	g				x	
2537946	g	a		x			
2545054	c	t				x	
2842022	c	t				x	
2949554	a	g				x	
3221165	a	g				x	
3839830	t	c		x			
4054862	t	c		x			
4778002	t	c				x	
4798644	a	c				x	
5100000	c	t				x	
5165931	g	c				x	

Table S4. Single-nucleotide polymorphisms that distinguish strain X13 from strain 66b.

Position on GenBank: AM039952.1	Reference allele	Alternative allele	**66b**	Tu-10	**X13**	X22	X31
8597	g	t			x		
76591	c	g			x		
394769	t	c	x				
649516	a	c	x				
876873	c	a	x				
893962	g	c			x		
928172	t	c			x		
980425	a	g			x		
1227342	t	g	x				
1281625	t	a			x		
1297319	g	a	x				
1384282	a	c			x		
1442912	g	a	x				
1505099	g	a	x				
1590365	c	t			x		
1600893	g	t	x				
1860694	t	g	x				
1937698	t	g			x		
2169318	a	c			x		
2371313	g	t	x				
2827142	g	a			x		
2827143	c	g			x		
2888596	t	c			x		
2894479	c	a	x				
2929910	g	a			x		
2966894	c	g			x		
3025998	t	a	x				
3623235	t	c	x				
3626498	t	c	x				
3770912	g	a	x				
4278932	g	t	x				
4278933	t	g	x				
4375039	c	t	x				
4426191	t	c	x				
4673860	c	t	x				
4761790	c	g	x				
4810267	g	t	x				
5030625	g	a	x				

5054504	c	t	x				
5157251	t	c			x		

Table S5. Single-nucleotide polymorphisms that distinguish strain X22 from strain X31.

Position on GenBank: AM039952.1	Reference allele	Alternative allele	66b	Tu-10	X13	**X22**	**X31**
74213	t	c				x	
512211	a	g				x	
809587	a	g				x	
861129	a	c					x
995341	t	c				x	
1018308	g	c				x	
1788346	t	g					x
1805242	t	c					x
1961368	g	a				x	
2169416	a	g					x
2172890	a	t				x	
2387221	a	g				x	
2545054	c	t				x	
2842022	c	t				x	
2934251	c	g					x
2949554	a	g				x	
3205571	g	a					x
3221165	a	g				x	
4778002	t	c				x	
4798644	a	c				x	
5100000	c	t				x	
5165931	g	c				x	

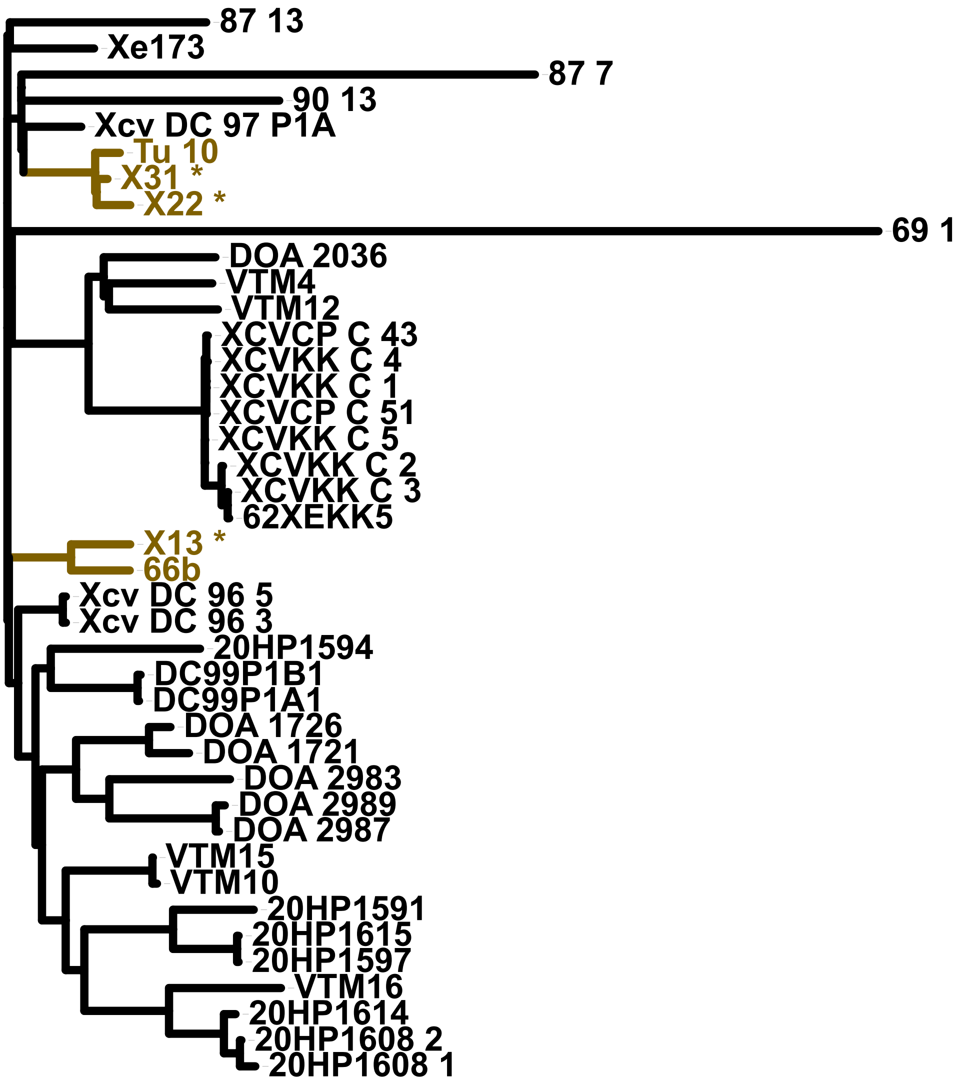

Figure S1. Phylogenetic position of strains X13, X22 and X31 within Clade 6 of *X. euvesicatoria* pv. *euvesicatoria* The tree is based on core genome sequences, generated using PhaME [11] and FastTree 2 [12]. In addition to the genomes of three Serbia strains, the tree also includes previously published genome sequences, whose accession numbers are listed in Supplementary Table S1. The tree was graphically rendered using the Interactive Tree of Life [13].

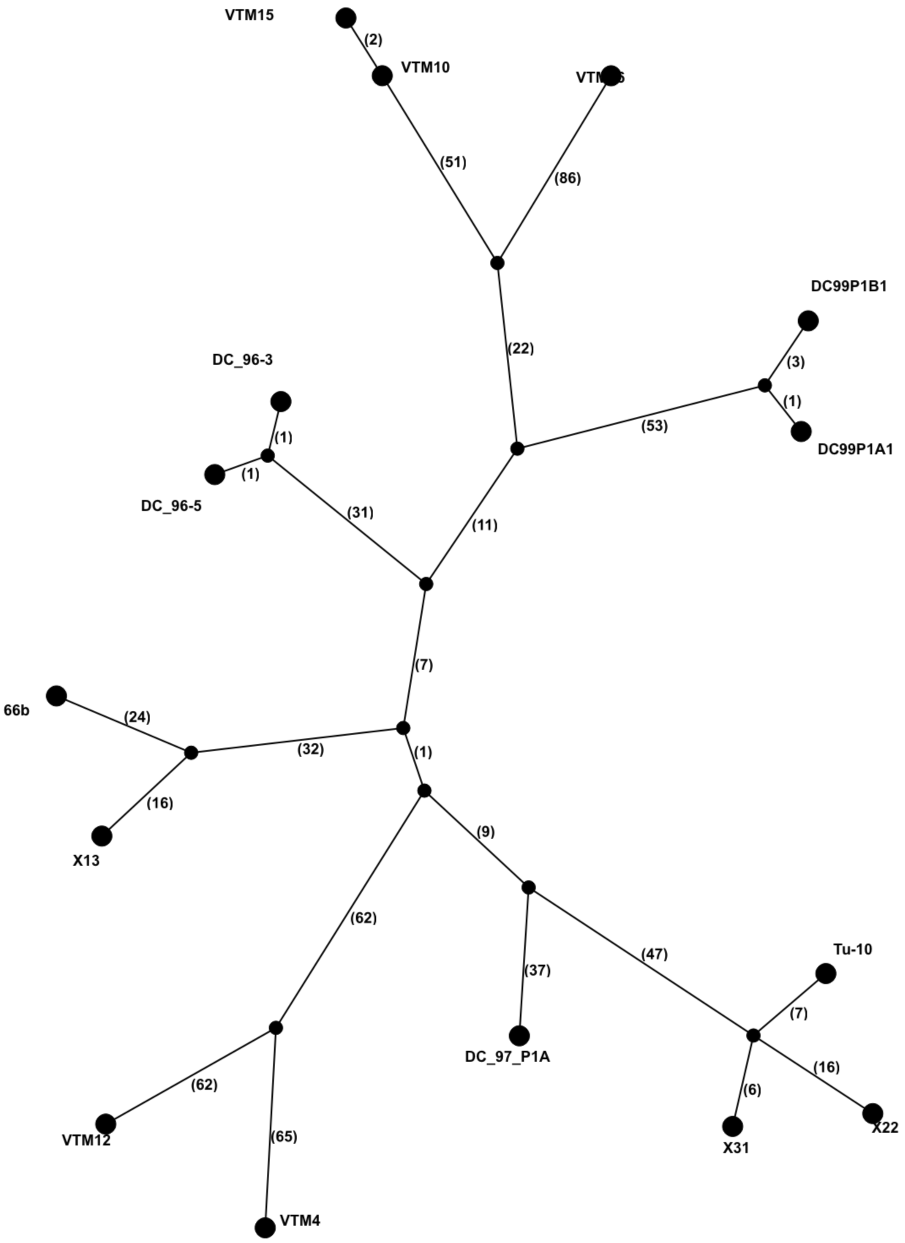

Figure S2. TCS network, created with PopArt for *X. euvesicatoria* pv. *euvesicatoria* strains in Clade 6. The numbers of single-nucleotide polymorphisms (SNPs) separating each node are indicated on the edges. The network is based on SNPs called against the 85-10 reference genome sequence.

A

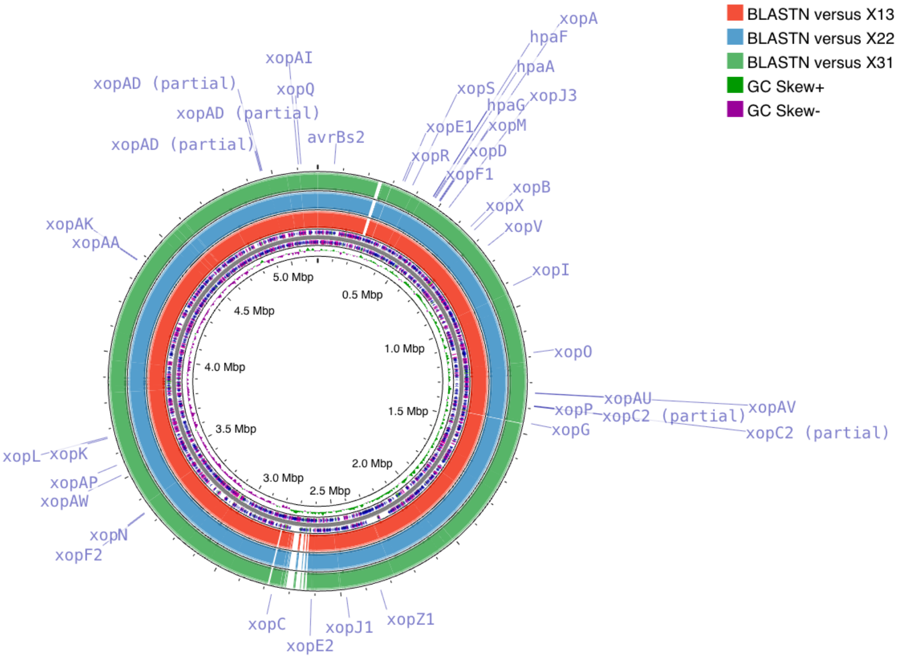

B

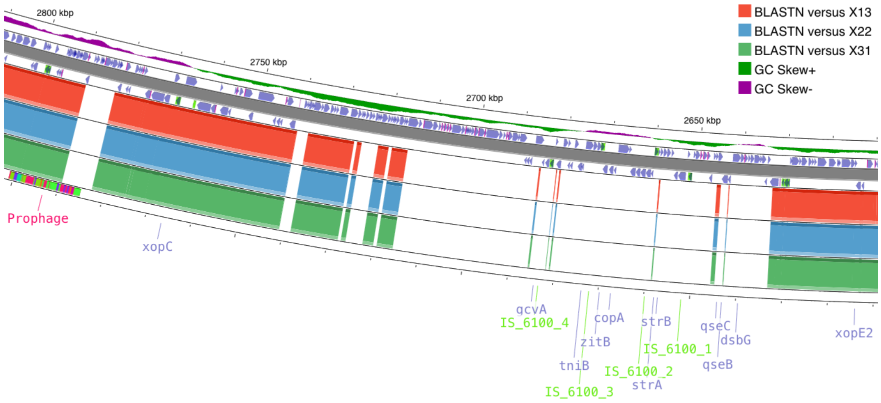

Figure S3. Comparison of Serbian *X. euvesicatoria* pv. *euvesicatoria* strains against the chromosome of reference strain 85-10. In Panel **A** the sequence of the 85-10 chromosome (GenBank: AM039952.1) is represented as a circle. Sequence similarity to X13, X22 and X31 genome sequences, identified through BLASTN searches, is indicated by the colouring of concentric circular tracks. The BLASTN searches were performed and visualised using Proksee web server [14]. Panel B is zoomed in to the region around the *xopC* and *xopE* genes, to more clearly illustrate the regions of the *Xeu* 85-10 chromosome that are absent from the Serbian *Xeu* strains.

A

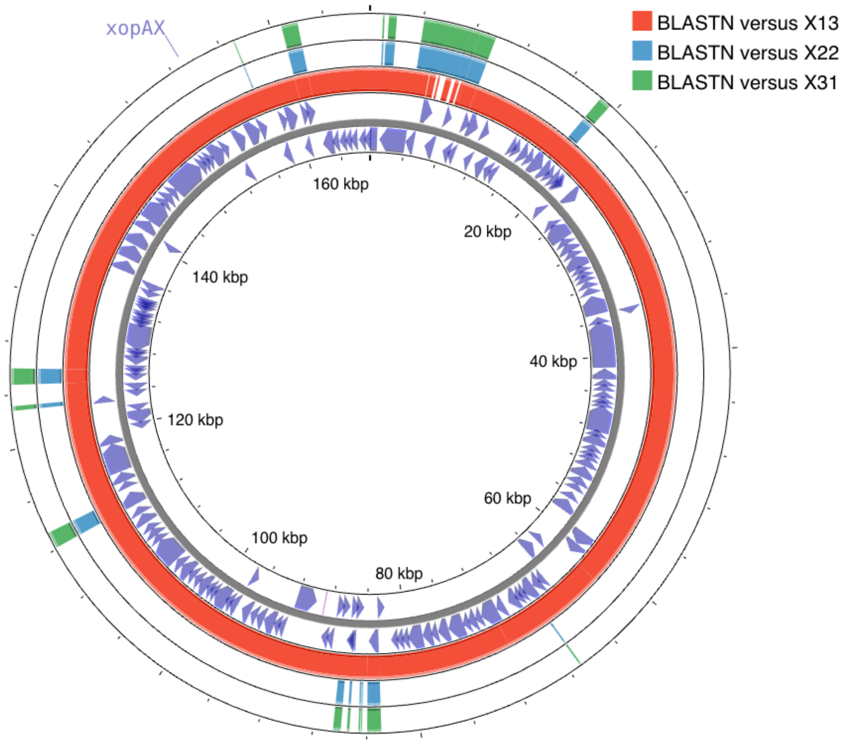

B

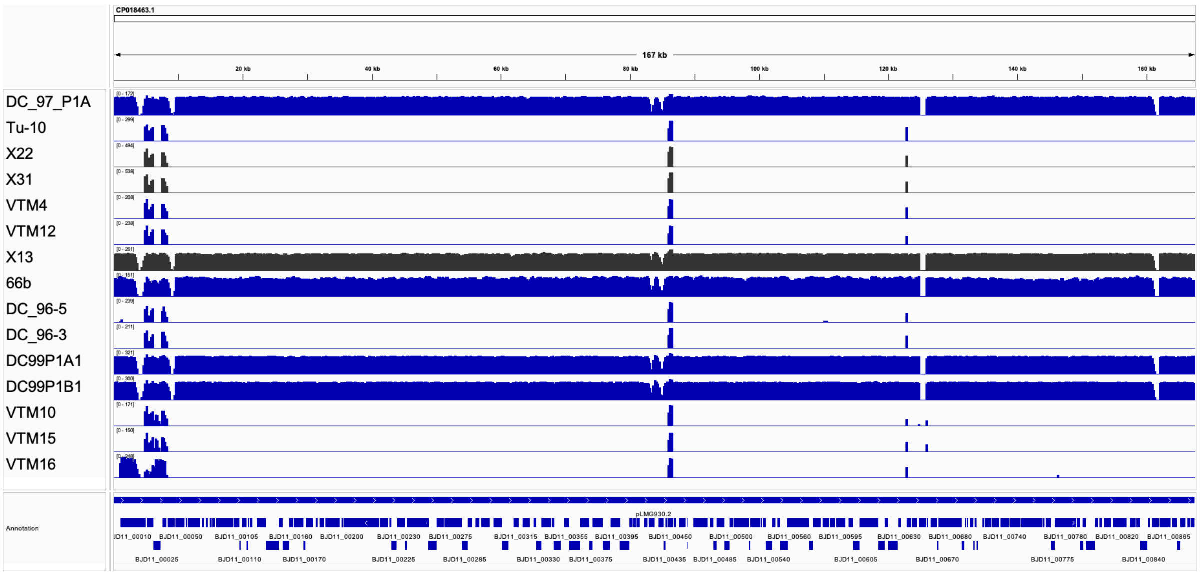

Figure S4. Strain X13 shares extensive sequence similarity with pXCV183-like plasmid pLMG930.2. Panel **A**: The sequence of the plasmid pLMG930.2 (GenBank: CP018463.1) is represented as a circle. Sequence similarity to *Xeu* genome sequences, identified through BLASTN searches, is indicated by the colouring of concentric circular tracks. The BLASTN searches were performed and visualised using Proksee web server [14]. Panel **B**: An alignment of genomic sequence reads from the 15 *Xeu* strains of Clade 6 versus plasmid pLMG930.2. The alignment was performed using BWA-mem [15] and a plot of coverage was visualized with the Integrative Genome Viewer (IGV) [16].

A

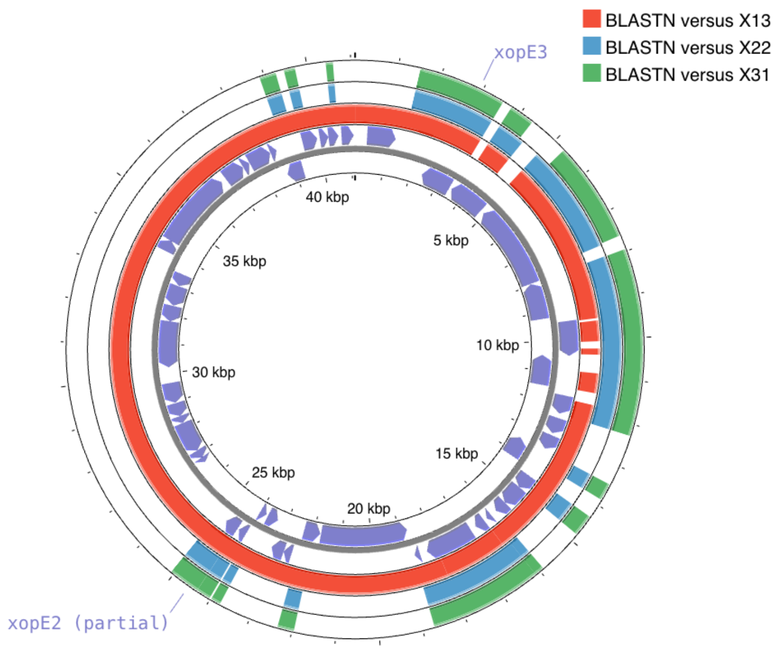

B

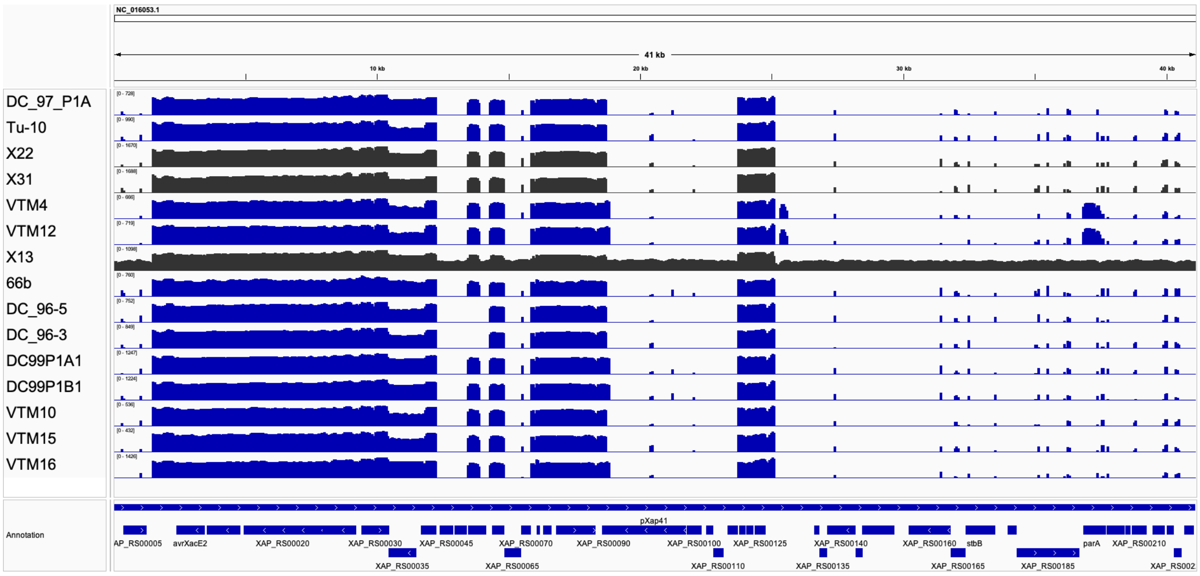

Figure S5. Strain X13 shares extensive sequence similarity with plasmid pXap41. The sequence of the *Xanthomonas arboricola* pv. *pruni* CFBP 5530 plasmid pXap41 (RefSeq: NC_016053.1) is represented as a circle. Sequence similarity to *Xeu* genome sequences, identified through BLASTN searches, is indicated by the colouring of concentric circular tracks. The BLASTN searches were performed and visualised using Proksee web server [14]. Panel **B**: An alignment of genomic sequence reads from the 15 *Xeu* strains of Clade 6 versus plasmid pXap41. The alignment was performed using BWA-mem [15] and a plot of coverage was visualized with the Integrative Genome Viewer (IGV) [16].

A

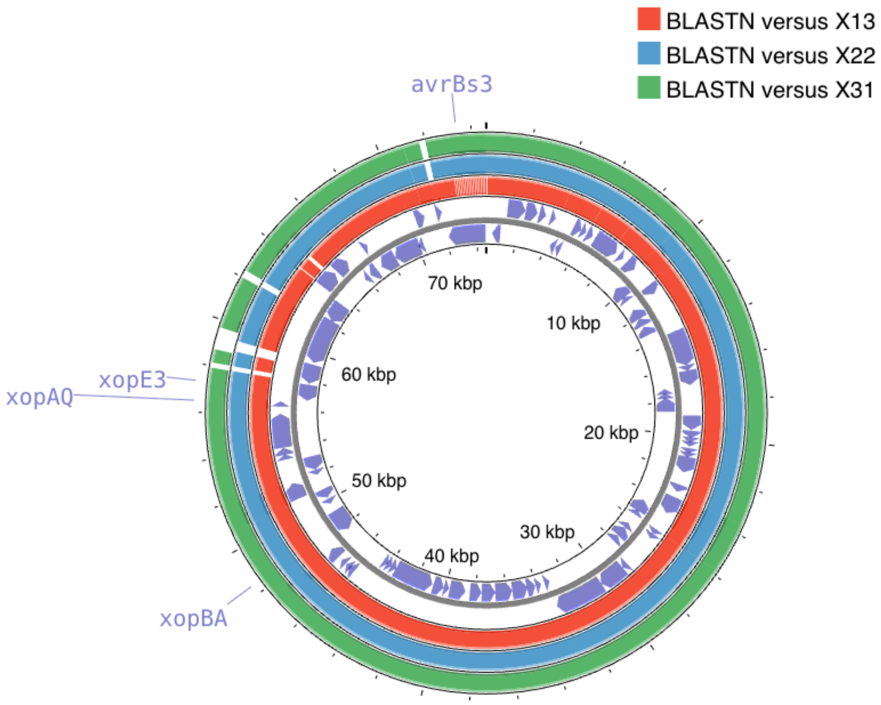

B

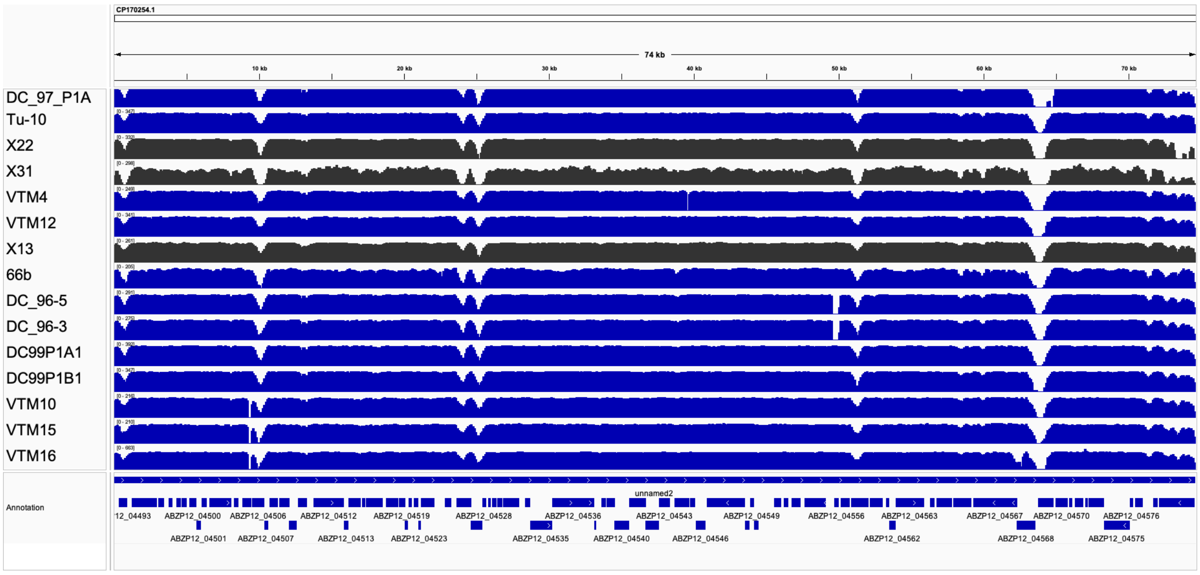

S6. Strains X13, X22 and X31 share extensive sequence similarity with a 75-kbp plasmid from strain Xe173. The sequence of the plasmid (GenBank: CP170254.1) is represented as a circle. Sequence similarity to *Xeu* genome sequences, identified through BLASTN searches, is indicated by the colouring of concentric circular tracks. The BLASTN searches were performed and visualised using Proksee web server [14]. Panel **B**: An alignment of genomic sequence reads from the 15 *Xeu* strains of Clade 6 versus the 75-kb plasmid. The alignment was performed using BWA-mem [15] and a plot of coverage was visualized with the Integrative Genome Viewer (IGV) [16].

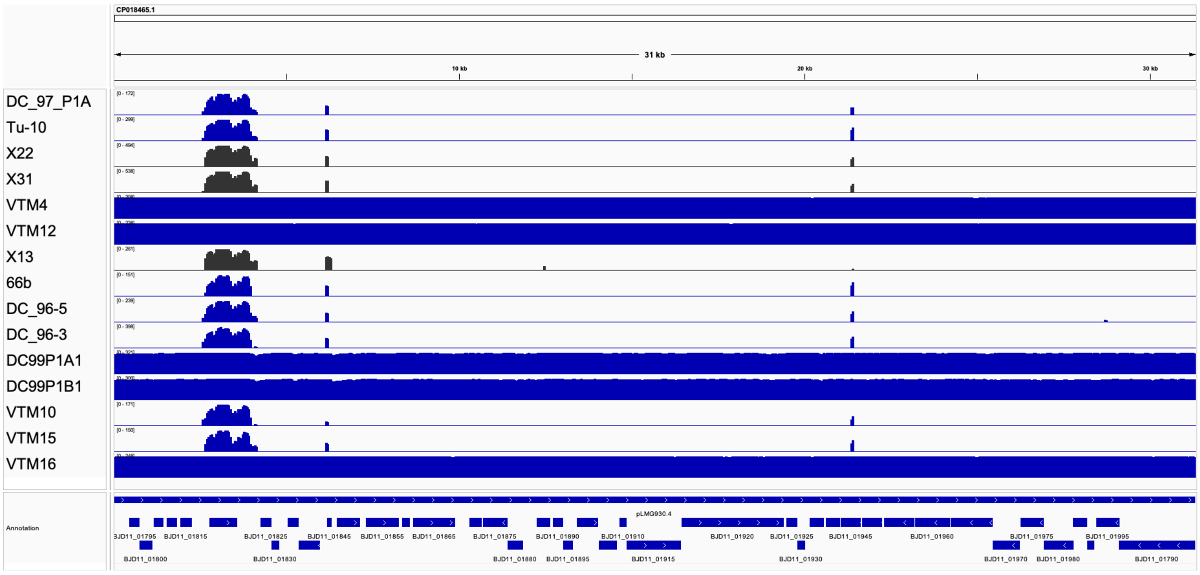

Figure S7. Strains X13, X22 and X31 do not share extensive sequence similarity with 31-kb plasmid pLMG930.4. An alignment of genomic sequence reads from the 15 *Xeu* strains of Clade 6 versus plasmid pLMG930.4 (GenBank: CP018465.1). The alignment was performed using BWA-mem [15] and a plot of coverage was visualized with the Integrative Genome Viewer (IGV) [16].

References

1. Tambong, J.T.; Xu, R.; Cuppels, D.; Chapados, J.; Gerdis, S.; Eyres, J.; Koziol, A.; Dettman, J. Whole-Genome Resources and Species-Level Taxonomic Validation of 89 Plant-Pathogenic *Xanthomonas* Strains Isolated from Various Host Plants. *Plant Disease* **2022**, *106*, 1558–1565, doi:10.1094/PDIS-11-21-2498-SC.
2. Barak, J.D.; Vancheva, T.; Lefeuvre, P.; Jones, J.B.; Timilsina, S.; Minsavage, G.V.; Vallad, G.E.; Koebnik, R. Whole-Genome Sequences of Xanthomonas Euvesicatoria Strains Clarify Taxonomy and Reveal a Stepwise Erosion of Type 3 Effectors. *Frontiers in Plant Science* **2016**, *7*, doi:10.3389/fpls.2016.01805.
3. Vancheva, T.; Lefeuvre, P.; Bogatzevska, N.; Moncheva, P.; Biology, F.; Kliment, S.; Montpellier, U.M.R.I. Draft Genome Sequences of Two Xanthomonas Euvesicatoria Strains from the Balkan Peninsula. **2015**, *3*, 6–7, doi:10.1128/genomeA.01528-14.
4. Parajuli, A.; Subedi, A.; Timilsina, S.; Minsavage, G.V.; Kenyon, L.; Chen, J.-R.; Goss, E.M.; Paret, M.L.; Jones, J.B. Phenotypic and Genetic Diversity of Xanthomonads Isolated from Pepper (Capsicum Spp.) in Taiwan from 1989 to 2019. *Phytopathology* **2024**, *114*, 2033–2044, doi:10.1094/PHYTO-11-23-0449-R.
5. Kaur, A.; Minsavage, G.V.; Potnis, N.; Jones, J.B.; Goss, E.M. Evolution of Copper Resistance in *Xanthomonas Euvesicatoria* Pv. *Perforans* Population. *mSystems* **2024**, e01427-24, doi:10.1128/msystems.01427-24.
6. Preangtong, Y.; Patarapuwadol, S.; Phiriyangkul, P.; Kanhayart, T.; Kositcharoenkul, N.; Kositratana, W.; Watcharachaiyakup, J. Characterisation and Genomic Diversity of *Xanthomonas* Species Causing Bacterial Spot Disease of Tomato and Pepper in Thailand. *Plant Pathology* **2025**, *74*, 1315–1334, doi:10.1111/ppa.14094.

7. Subedi, A.; Kara, S.; Aysan, Y.; Minsavage, G.V.; Timilsina, S.; Roberts, P.D.; Goss, E.M.; Jones, J.B. Draft Genome Sequences of 11 Xanthomonas Strains Associated with Bacterial Spot Disease in Turkey. *Access Microbiol* **2023**, *5*, acmi000586.v3, doi:10.1099/acmi.0.000586.v3.
8. Thieme, F.; Koebnik, R.; Bekel, T.; Berger, C.; Boch, J.; Büttner, D.; Caldana, C.; Gaigalat, L.; Goesmann, A.; Kay, S.; et al. Insights into Genome Plasticity and Pathogenicity of the Plant Pathogenic Bacterium Xanthomonas Campestris Pv. Vesicatoria Revealed by the Complete Genome Sequence. *Journal of bacteriology* **2005**, *187*, 7254–7266, doi:10.1128/JB.187.21.7254-7266.2005.
9. Richard, D.; Boyer, C.; Lefeuvre, P.; Canteros, B.I.; Beni-Madhu, S.; Portier, P.; Pruvost, O. Complete Genome Sequences of Six Copper-Resistant Xanthomonas Strains Causing Bacterial Spot of Solaneous Plants, Belonging to X. Gardneri, X. Euvesicatoria, and X. Vesicatoria, Using Long-Read Technology. *Genome announcements* **2017**, *5*, doi:10.1128/genomeA.01693-16.
10. Subedi, A.; Nga, N.T.T.; Tien, D.T.K.; Minsavage, G.V.; Roberts, P.D.; Goss, E.M.; Jones, J.B. Draft Genomes Announcement of Vietnamese Xanthomonas Euvesicatoria Strains Causing Bacterial Spot on Pepper. *Access Microbiol* **2024**, *6*, 000741.v3, doi:10.1099/acmi.0.000741.v3.
11. Shakya, M.; Ahmed, S.A.; Davenport, K.W.; Flynn, M.C.; Lo, C.-C.; Chain, P.S.G. Standardized Phylogenetic and Molecular Evolutionary Analysis Applied to Species across the Microbial Tree of Life. *Scientific reports* **2020**, *10*, 1723, doi:10.1038/s41598-020-58356-1.
12. Price, M.N.; Dehal, P.S.; Arkin, A.P. FastTree 2 - Approximately Maximum-Likelihood Trees for Large Alignments. *PLoS ONE* **2010**, *5*, doi:10.1371/journal.pone.0009490.
13. Letunic, I.; Bork, P. Interactive Tree Of Life (iTOL) v5: An Online Tool for Phylogenetic Tree Display and Annotation. *Nucleic Acids Research* **2021**, *49*, W293–W296, doi:10.1093/nar/gkab301.
14. Grant, J.R.; Enns, E.; Marinier, E.; Mandal, A.; Herman, E.K.; Chen, C.-Y.; Graham, M.; Van Domselaar, G.; Stothard, P. Proksee: In-Depth Characterization and Visualization of Bacterial Genomes. *Nucleic Acids Res* **2023**, *51*, W484–W492, doi:10.1093/nar/gkad326.
15. Li, H. Aligning Sequence Reads, Clone Sequences and Assembly Contigs with BWA-MEM. **2013**, 3.
16. Robinson, J.T.; Thorvaldsdóttir, H.; Winckler, W.; Guttman, M.; Lander, E.S.; Getz, G.; Mesirov, J.P. Integrative Genomics Viewer. *Nature biotechnology* **2011**, *29*, 24–26, doi:10.1038/nbt.1754.
